# Proteomics and Molecular Docking Analyses Reveal the Bio-Chemical and Molecular Mechanism Underlying the Hypolipidemic Activity of Nano-Liposomal Bioactive Peptides in 3T3-L1 Adipocytes

**DOI:** 10.3390/foods12040780

**Published:** 2023-02-10

**Authors:** Sucheewin Krobthong, Yodying Yingchutrakul, Patompon Wongtrakoongate, Hathaichanok Chuntakaruk, Thanyada Rungrotmongkol, Chartchai Chaichana, Thanisorn Mahatnirunkul, Thitikorn Chomtong, Kiattawee Choowongkomon, Chanat Aonbangkhen

**Affiliations:** 1 Center of Excellence in Natural Products Chemistry (CENP), Department of Chemistry, Faculty of Science, Chulalongkorn University, Bangkok 10330, Thailand; 2 Center for Neuroscience, Faculty of Science, Mahidol University, Bangkok 10400, Thailand; 3 National Omics Center, NSTDA, Pathum Thani 12120, Thailand; 4 Department of Biochemistry, Faculty of Science, Mahidol University, Bangkok 10400, Thailand; 5 Center of Excellence in Biocatalyst and Sustainable Biotechnology, Department of Biochemistry, Faculty of Science, Chulalongkorn University, Bangkok 10330, Thailand; 6 Program in Bioinformatics and Computational Biology, Graduate School, Chulalongkorn University, Bangkok 10330, Thailand; 7 Siriraj Center of Research Excellence for Diabetes and Obesity (SiCORE-DO), Faculty of Medicine Siriraj Hospital, Mahidol University, Bangkok 10700, Thailand; 8 National Nanotechnology Center, NSTDA, Pathum Thani 12120, Thailand; 9 Department of Biochemistry, Faculty of Science, Kasetsart University, Bangkok 10900, Thailand

**Keywords:** liposome, nanoparticles, adipocyte, glycerol, lipolysis, orlistat, HDOCK, fatty acid synthase

## Abstract

Obesity is a global health concern. Physical activities and eating nutrient-rich functional foods can prevent obesity. In this study, nano-liposomal encapsulated bioactive peptides (BPs) were developed to reduce cellular lipids. The peptide sequence NH_2_-PCGVPMLTVAEQAQ-CO_2_H was chemically synthesized. The limited membrane permeability of the BPs was improved by encapsulating the BPs with a nano-liposomal carrier, which was produced by thin-layer formation. The nano-liposomal BPs had a diameter of ~157 nm and were monodispersed in solution. The encapsulation capacity was 61.2 ± 3.2%. The nano-liposomal BPs had no significant cytotoxicity on the tested cells, keratinocytes, fibroblasts, and adipocytes. The in vitro hypolipidemic activity significantly promoted the breakdown of triglycerides (TGs). Lipid droplet staining was correlated with TG content. Proteomics analysis identified 2418 differentially expressed proteins. The nano-liposomal BPs affected various biochemical pathways beyond lipolysis. The nano-liposomal BP treatment decreased the fatty acid synthase expression by 17.41 ± 1.17%. HDOCK revealed that the BPs inhibited fatty acid synthase (FAS) at the thioesterase domain. The HDOCK score of the BPs was lower than that of orlistat, a known obesity drug, indicating stronger binding. Proteomics and molecular docking analyses confirmed that the nano-liposomal BPs were suitable for use in functional foods to prevent obesity.

## 1. Introduction

Bioactive peptides (BPs) are composed of protein fragments or peptides with beneficial metabolic and physiological functions to promote human health [1,2]. BPs are found in natural sources and hydrolyzed parent proteins. Most BPs have similar structures such as lengths of <20 amino acid residues and contain hydrophobic residues [3,4]. Based on the mode of action, BPs are classified as anti-microbial, anti-angiotensin converting enzyme, anti-oxidative stress, anti-thrombotic, immunomodulatory, and anti-hypolipidemic peptides [5,6,7,8,9,10]. Hyperlipidemia is directly associated with obesity, which is caused by the excess accumulation of triacylglycerol in adipocytes. The increased breakdown or hypolipidemic activity of triacylglycerol may contribute to reducing the fat and triglyceride (TG) contents of the cells and tissues. Many peptides originating from natural sources can be incorporated into foods for effective delivery [4]. BPs, as a new generation of functional food ingredients, can be used to administer ingredients to prevent and treat non-infectious diseases and various metabolic diseases such as hypertension, diabetes, and obesity. BPs carrying the tripeptide LKP have antihypertensive activities [11] and the hexapeptide VDLPTC derived from *Lingzhi* (*Ganoderma lucidum*, Reishi mushroom) exhibits radical scavenging activities [5]. BPs with health-promoting effects can be identified, purified, modified, and developed into a variety of commercial food ingredients with few adverse effects in a cost-effective and environmentally friendly manner [12]. Generally, BPs have low membrane permeability due to relatively high water solubility due to the amide backbone and sidechains [13]. After oral administration, BPs cross the biological membrane into the circulation to access the target molecules. Encapsulation of BPs with a carrier can enhance transportation efficiency and resistance to proteases.

The utilization of BPs in various biological applications has started to emerge. Proteins and peptides are generally not considered as good functional ingredient candidates compared with small molecules due to the limited cell membrane permeability. However, different types of vesicular carrier systems can improve the transport of BPs across the cell membrane. Liposomes are self-forming lipid bilayer structures with separate hydrophilic and hydrophobic regions that enable the encapsulation of water-soluble compounds in the aqueous core region [14]. Encapsulation in liposomes protects BPs from proteases while improving the membrane permeability and prolonging the biological effects. Hence, the potential usefulness of liposomes as carriers for BPs has attracted considerable interest from manufacturers of functional foods.

Our group has previously reported that protein hydrolysates derived from *Lingzhi*, as a functional food ingredient, prevented oxidative stress and lipid accumulation [6,15]. However, the ability of the specific BP sequence NH_2_-PCGVPMLTVAEQAQ-CO_2_H to reduce lipid accumulation in adipocytes and the underlying mechanisms remain unclear. Hence, liposome-based (nano-liposomal) BPs that retain the beneficial effects with high membrane permeability were established as a functional food additive.

## 2. Materials and Methods

### 2.1. Peptide Synthesis and Purity and Deviation Mass Evaluation

The peptide NH_2_-PCGVPMLTVAEQAQ-CO_2_H was produced by solid-phase peptide synthesis (SPPS) in a 15-mL filter funnel (Z283304; Sigma-Aldrich Corporation, St. Louis, MO, USA) and established on Rink Amide AM resin (Bachem AG, Bubendorf, Switzerland). The synthesis reaction is described in Supplementary material.

The dried synthetic peptide was solubilized in 0.1% formic acid/water. The purity of the synthetic peptide was measured by high performance liquid chromatography (HPLC) as the peak area of the peptide at a wavelength of 220 nm, while the peptide mass was determined by liquid chromatography-mass spectrometry (LC-MS) as the difference between the theoretical and observed masses. The synthesized products were separated with an Inertsil^®®^ ODS-3 analytical column (particle size, 3 μm; inner diameter, 4.6 mm; length, 250 mm) with a linear gradient of mobile phase A (0.065% trifluoroacetic acid (TFA) in water) and mobile phase B (0.05% TFA in CH_3_CN) at a flow rate of 1 mL/min for 40 min. The peptide mass measurement deviation was calculated using the following equation:

$$\mathrm{Molecular}\;\mathrm{mass}\;\mathrm{deviation}\;\left(\%\right)=\frac{\Delta \mathrm{mass}}{\mathrm{Theoretical}\;\mathrm{mass}}\;\times \;100\%$$

where Δmass is the observed peptide mass as determined by LC-MS (experimental mass), and the theoretical mass is calculated from the masses of the constituent atoms.

### 2.2. Preparation and Characterization of Nano-Liposomal BPs by Thin-Film Hydration

Nano-liposomes for the encapsulation of BPs were established using a thin-film formation approach [15]. Lecithin and cholesterol were dissolved in 16 mL of diethyl ether in a 50-mL round-bottom flask and then evaporated in a vacuum rotary evaporator at 90–100 rpm under reduced pressure. BPs in phosphate-buffered saline (PBS) solution were added to 10 mL of thin-film and agitated with an orbital shaker at 220 rpm for 6 h at 27–28 °C. The liposomal/BP suspension was extruded 25 times through a 200-nm pore size polycarbonate membrane using an Avanti mini extruder (Avanti Polar Lipids, Inc., Alabaster, AL, USA) to produce particles with similar sizes. The nano-liposomal BPs were collected by ultracentrifugation at 100,000× *g* for 120 min at 4 °C. The nano-liposomal pellet was suspended in PBS prior to the measurement of the loading efficiency, size distribution, and poly-dispersity index (PdI).

The BP encapsulation efficiency of the nano-liposomal carrier was determined by protein content-based spectrophotometry. In brief, 100 µL of nano-liposomal BPs were mixed with 2% Triton X-100 (1:1, *v*/*v*) and sonicated (at 65% maximum amplitude) for 10 min (20-s intervals) to disassemble the liposomes and release the BPs. Afterward, the total protein content of the solution was evaluated using the Lowry protein assay using bovine serum albumin as a protein reference. The loading efficiency was calculated using the following equation:

$$\mathrm{BP}\;\mathrm{loading}\;\mathrm{efficiency}\;\left(\%\right)=\frac{[\mathrm{BP}\;\mathrm{extracted}\;\mathrm{of}\;\mathrm{which}\;\mathrm{encapsulated}\;\mathrm{in}\;\mathrm{liposomes}}{\mathrm{Total}\;\mathrm{BP}\;\mathrm{content}}\;\times \;100\%$$

The size distribution and PdI of the nano-liposomal BPs were determined by dynamic light scattering (photo correlation spectroscopy) using a ZetaSizer Nano-ZS device (Malvern Instruments, Malvern, Worcestershire, UK). The nano-liposomal BPs were diluted before analysis with deionized water. The particle size distribution and PdI were measured at 25 °C. The size distribution and PdI of nanoliposome BPs were determined by dynamic light scattering using ZetaSizer Nano-ZS devices.

### 2.3. Cell Cytotoxicity of Nano-Liposomal BPs

Immortalized human keratinocytes (HaCaT cells), human fibroblasts, and mouse differentiated adipocytes (3T3-L1 cells) were induced with an adipogenic cocktail (2.5 mM dexamethasone, 0.5 mM 3-isobutyl-1-methylxanthine, and 10 g/mL of insulin) to assess the cell cytotoxicity of the nano-liposomal BPs as a treatment group at various concentrations (12.5, 6.25, 3.125, 1.563, 0.781, 0.391, 0.195, 0.097, 0.049, and 0.244 µg/mL) with unloaded nano-liposomal BPs as a control group. The MTT (3-[4,5-dimethylthiazol-2-yl]-2,5 diphenyl tetrazolium bromide) assay was used to calculate the percentage of viable cells after 12 h of treatment with the no-observed-adverse-effect level (NOAEL) to study the effect of nano-liposomal BPs using a proteomics approach.

### 2.4. In Vitro Hypolipidemic Activity of Nano-Liposomal BP

Mouse adipocytes were used to determine the in vitro hypolipidemic activity. In brief, the content of glycerol, a by-product of lipolysis, in the culture medium of loaded and unloaded nano-liposomal BPs was measured using an EnzyChrom™ Glycerol Assay Kit (Bioassay Systems, Hayward, CA, USA). To evaluate the intracellular TG content, the treated cells were washed twice with PBS, and fixed with 4% paraformaldehyde for 30 min at room temperature. The fixed cells were washed with 60% isopropanol and stained with 0.5% Oil Red O (ORO) in 60% isopropanol for 1 h at room temperature. To avoid nonspecific staining, the cells were rinsed with deionized water. Lipid droplets were observed under a stereomicroscope at 20× magnification. The cellular lipid droplets were dissolved in isopropanol for 15 min prior to the measurement of the TG content with a microplate reader (Multiskan™ GO; Thermo Fisher Scientific, Waltham, MA, USA) at a wavelength of 510 nm. Unloaded nano-liposomes were used as the control.

### 2.5. Label-Free Proteomic Analysis, Quality Control (QC), and Bioinformatics Analysis

To investigate the adipocyte proteome profiles of 3T3-L1 cells after exposure to the nano-liposomal BPs, the treated cells were lysed as described in a previous study [15]. The lysed cells were cleaned-up and digested with trypsin (Promega Corporation, Madison, WI, USA) at an enzyme:protein ratio of 1:40 and incubated a 37 °C for 4 h. The tryptic peptides were dried and reconstituted in 0.1% formic acid/water to obtain 500 ng/μL of tryptic peptides, which were further analyzed by LC-MS/MS.

The LC-MS/MS spectrum data were collected in the positive mode with an HF Hybrid Quadrupole-Orbitrap™ Mass Spectrometer combined with a nano-LC system equipped with a nano C18 column. Mobile phase A consisted of 0.1% formic acid in water and mobile phase B consisted of 90% acetonitrile with 0.1% formic acid. The samples were loaded directly onto an analytical C_18_ column. Separation was conducted with a linear gradient of 2–45% mobile phase B at a constant flow rate of 300 nL/min over a period of 135 min. The analytical column was regenerated with 90% mobile phase B for 10 min and re-equilibrated with 5% mobile phase B for 35 min. The tryptic peptides were analyzed by applying a data-dependent acquisition method, followed by a higher-energy collisional dissociation (collision energy = 28 eV). Full scan mass spectra were acquired at an *m*/*z* ratio of 400 to 1600 with an AGC target set at 3 × 10^6^ ions and a resolution of 120 k. MS/MS scanning was initiated when the automatic gain control target reached 1 × 10^5^ ions and a resolution of 15 k. The raw mass spectra (raw file) were processed by Proteome Discoverer™ 2.4 software (Thermo Fisher Scientific, Waltham, MA, USA) and identified against the UniProt protein database (https://www.uniprot.org/ (accessed on 15 December 2022); organism: *Mus musculus*; 20,395 sequences). Protein identification and quantification were carried out with the following parameters: MS tolerance (20 ppm); MS/MS tolerance (0.05 Da); digestion enzyme (trypsin); fixed modification (cysteine carbamidomethylation); and variable modification (methionine oxidation). The detection rate of peptides and the proteins’ false discovery rate (FDR) were set to 1%. The relative protein abundance was standardized using the software’s normalization algorithm (total amount of peptides). QC included variation in total ion count and peptide–spectrum match efficiency were determined. The FDR was used to estimate the number of peptide–spectrum matches. Data that passed the QC were subjected to downstream analysis. The raw files were deposited at the ProteomeXchange Consortium proteomics resource (http://www.proteomexchange.org) (accessed on 15 December 2022) via the PRIDE partner repository with the dataset identifiers.

To assess the biological and technical heterogeneity, principal component analysis (PCA) was used to visualize the differences between group replications within and between groups. Gene Ontology (GO) enrichment analysis was performed manually using web-based software tools and the UniProt database (http://www.uniprot.org/uniprot) (accessed on 15 December 2022).

### 2.6. Quantification of Fatty Acid Synthase (FAS) Using an Enzyme-Linked Immunosorbent Assay (ELISA) for Proteomics Validation

After treatment with nano-liposomal BPs, the 3T3-L1 cells were lysed and FAS activity was measured using a sandwich ELISA Kit (LSF7308; LSBio Co., Seattle, WA, USA) with a 96-well plate format. The lysates of treated and untreated cells were cleaned-up using Zeba™ spin desalting columns and diluted with sample diluent buffer to obtain a final concentration of 3.125 μg/mL. Then, 100 μL aliquots of the samples were added to the wells of 96-well plates and incubated for 2 h at 37 °C (n = 3). Afterward, 100 μL of Detection reagent A was added to each well and the plate was incubated for 2 h at 37 °C. Next, the solution was discarded and the plate was washed five times with 1× wash buffer. Afterward, 100 μL of Detection reagent B were added to each well and the plate was incubated for 40 min at 37 °C. Then, the solution was discarded and the plate was washed seven times with 1× wash buffer. To determine the level of FAS expression, 100 μL of 3, 3’, 5, 5’-tetramethylbenzidine substrate solution was added and the plate was incubated for 25 min at 37 °C. The reaction was terminated by the addition of 50 μL of stopping solution. Finally, the absorbance was measured with a microplate reader at a wavelength of 450 nm. The expression level was calculated according to the following equation:

$$\%\mathrm{enzyme}\;\mathrm{activity}=\frac{\left(\lambda_{control}-\lambda_{sample}\right)}{\lambda_{control}}\times 100\%$$

where Eλ_sample_ is the absorbance of the testing sample and λ_control_ is the absorbance of a blank control. The experiment was conducted in triplicate (n = 3).

### 2.7. Computational Studies of BP Binding to FAS Thioesterase

Molecular docking was performed to elucidate the mechanism of inhibition between the BP and the thioesterase (TE) domain of FAS. The three-dimensional (3D) structure of the protein target was predicted by AlphaFold 2 software (https://deepmind.com/research/case-studies/alphafold) (accessed on 9 September 2021) [16] using the protein sequence of FAS-TE (UniProtKB—P49327). Supplementary Figure S1A shows the number of sequences per position, the AlphaFold2 confidence measures (pLDDT), and all five models. The highest percentage of correctly predicted interatomic distances was 90.26% for the predicted structure Model1 of FAS TE. The focused BP structure with a sequence of NH_2_-PCGVPMLTVAEQAQ-CO_2_H was constructed by the PEP-FOLD3 (http://bioserv.rpbs.univ-paris-diderot.fr/services/PEP-FOLD3) (accessed on 9 September 2021) [17]. All five modeled BPs were in a helical structure (Supplementary Figure S1B). The coarse grained energy of PEP-FOLD3 (sOPEP), the predicted Global Distance Test Total Score (gdt), the predicted Qmean score (q), and the predicted Tm score (tm) suggest that Model1 is the best predicted structure of BP. Consequently, the resulting Model1 of both the peptide and protein was chosen for the docking study using the HDOCK server (http://hdock.phys.hust.edu.cn/) (accessed on 8 August 2021). The superimposition between the predicted structure and the crystal structure of the FAS TE/orlistat complex in chain B (PDB: 2PX6 [18]) was conducted with the University of California at San Francisco (UCSF) Chimera package (https://www.cgl.ucsf.edu/chimera/) (accessed on 17 November 2021) [19] to locate the binding site. The ionized states of the side chains were configured at pH 7.4 using PROPKA3.1 [20], while ChemAxon [8] was used to check the pKa value of the reference ligand bound to FAS TE, orlistat in the hydrolyzed form. The HDOCK score [21] of BP was calculated and compared with orlistat. The peptide–protein and ligand–protein binding were visualized using LigPlot [22].

### 2.8. Statistical Analysis

All experiments were conducted with at least three independent replicates (n = 3) and all data were expressed as the mean ± standard deviation (SD). The significance of differences was determined with Duncan’s multiple range test. All analyses were conducted using GraphPad Prism software version 5.0 (GraphPad Software, Inc., San Diego, CA, USA). The cell cytotoxicity comparison was analyzed using two-way analysis of variance followed by the Bonferroni post-test. A probability (*p*) value of <0.05 was considered statistically significant. The *p*-value was adjusted for multiple testing.

## 3. Results and Discussion

### 3.1. Peptide Synthesis Using SPPS Approach

The peptide sequence NH_2_-PCGVPMLTVAEQAQ-CO_2_H was produced by SPPS. The purity and molecular mass, as crucial QC parameters, were analyzed by HPLC and MS, respectively (Table 1).

The purity and mass accuracy were used to assess the similarity of the amino acid sequences of the synthesized and original peptides. The purity of the synthetic peptides was >85% and molecular mass reflected that the synthesis reaction was correct. However, the purity of the synthesized peptide was less than 100%, which could be due to many factors such as incomplete coupling, interruption of the amino acid side chains, and the cleavage efficiency of the synthesized peptide from the *N*-protecting group and the resin [23]. The inefficient deprotection of the amino acid side chain leads to unwanted peptide adducts. In SPPS, the oxidation of amino acid impurities was also observed [24]. The incomplete removal of solvent used in the peptide elongation step may lead to impurities such as substitution and addition products. In addition, some peptides that are poorly solvated while linked to the solid resin may prevent the completion of the reaction in the protection and coupling processes [25]. Nevertheless, the purity (>80%) of the synthetic peptides was appropriate to assess the biological activity in vitro [26]. Hence, the synthetic peptides were considered as a single compound to observe the lipolysis activity in cell-based studies.

### 3.2. Establishment and Characterization of the Nano-Carrier for BPs

The nano-liposome carrier established by thin-layer formation mostly encapsulated the BPs via the hydrophilic region. The encapsulation efficiency of the liposome carrier was estimated by the Lowry protein assay. However, various factors influence the loading efficiency including the type of lipid, molecular weight, charge, and molecule solubility [14]. The results revealed that the encapsulation efficiency was 61.17 ± 1.95%, indicating that the encapsulation capacity of the liposomal preparation was moderate. The BPs might interact with the lipid precursors to establish a liposome shell, which would disrupt entrapment of the BPs and prevent full loading capacity [27]. Alternatively, the electrostatic interactions among the side chains of the amino acid residues of the BPs and liposome surface could adversely affect the encapsulation efficiency.

The diameters of the loaded and unloaded nano-liposomal particles, as determined by dynamic light scattering, were 155.7 ± 0.37 and 134.2 ± 0.49 nm, respectively. The hydrodynamic size of the loaded liposome was at the nanoscale level, which was close to the ideal size (<0.2 μm) for the intended application, as particles must be sufficiently small to penetrate the membrane. As the efficiency of cellular uptake is related to particle size, a small particle size of <0.16 μm would have great potential for cellular uptake [28].

The PdI values of the loaded and unloaded nano-liposomal particles were relatively low at 0.09 ± 0.02 and 0.11 ± 0.003, respectively, indicating that the particles were monodispersed with a narrow size distribution and very high surface area ratio. This result also suggests the homogeneity of the liposomal particles in solution.

The zeta (ζ) potential is a measurement of the net charge of the particle surface that indicates stability in solution. The ζ potentials of the loaded and unloaded particles were −4.42 ± 0.02 and −5.74 ± 0.05 mV, respectively. The negative ζ potential value indicates that the particles are stable and will tend to repel each other and prevent aggregation, cellular fusion, and phagocytosis [29]. Therefore, the hydrodynamics of liposome size and negative ζ potential is an important factor that should be considered in various applications.

### 3.3. Cytotoxicity of the Loaded and Unloaded Nano-Liposomal Particles

**Figure 1:**
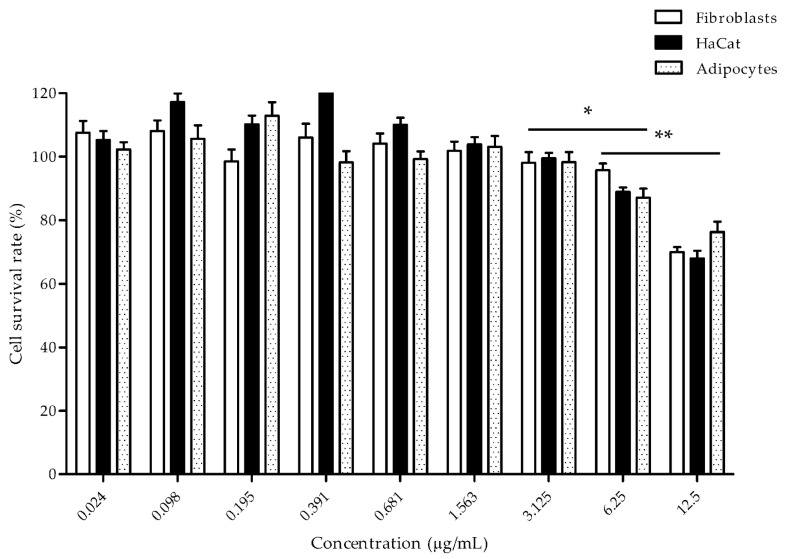
Cell cytotoxicity measured by the MTT assay. The cells were treated with increasing concentrations of nano-liposomal BPs for 24 h. Black, white, and dotted bars represent the differentiated HaCaT cells, fibroblasts, and adipocytes, respectively. The percentage of viable cells is shown on the *y* axis, while the concentrations of the nano-liposomal BPs are shown on the *x* axis (0.024–12.5 μg/mL). The data are presented as the mean ± SD. The symbols (*) and (**)indicate *p* < 0.05 and *p* < 0.01, respectively.

Cytotoxicity is an important concern when utilizing peptides in commercial products. Here, the cytotoxicity of three cell types (HaCaT cells, fibroblasts, and adipocytes) was tested by exposure to varying concentrations of the nano-liposomal BPs for 24 h using the MTT assay. The viability of the cells is illustrated in Figure 1.

The nano-liposomal BPs had no effect on cell viability, even at a high concentration of up to 3.125 µg/mL. At 12.5 µg/mL, the cells exhibited cytotoxic effects. At 6.25 µg/mL, the percentages of viable HaCaT cells, fibroblasts, and differentiated adipocytes were 88.97 ± 1.42%, 95.74 ± 2.11%, and 87.15 ± 2.84%, respectively. At the highest concentration, the percentages of viable HaCaT cells, fibroblasts, and differentiated adipocytes were 67.99 ± 2.42%, 69.98 ± 1.55%, and 76.32 ± 3.23%, respectively.

Various cell types were employed to evaluate the cytotoxicity of nano-liposomal BPs in vitro. The concentrations of the nano-liposomal BPs, as revealed by the MTT assay, had no detectable adverse effects up to 3.125 µg/mL, as defined as the NOAEL. The nano-liposomal BPs could be added to functional foods, thus potential cytotoxicity should be carefully considered. The NOAEL of the loaded liposomes was 3.125 μg/mL for the in vitro hypolipidemic experiments.

### 3.4. Effect of the Nano-Liposomal BP on Lipolysis Activity

**Figure 2:**
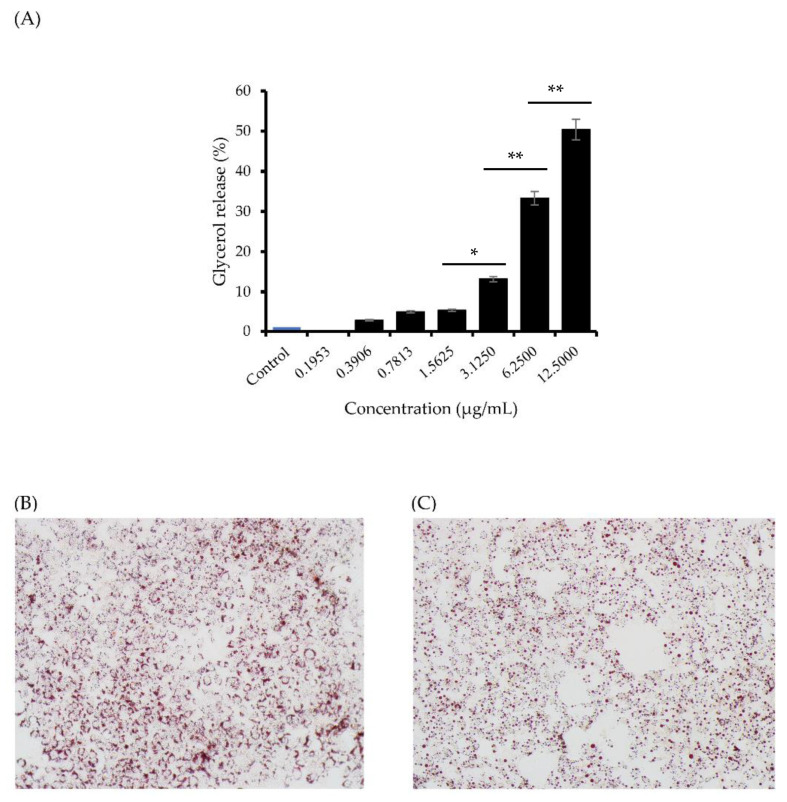
Effects of nano-liposomal BPs on lipid accumulation in 3T3-L1 cells. (**A**) The effect of nano-liposomal BP on glycerol release in the cells. The ORO-stained lipids were observed under a stereomicroscope (20× magnification). Cells stained with (**B**) a vehicle were used as the control and (**C**) the nano-liposomal BPs at 3.125 ug/mL. The symbols (*) and (**) indicate *p* < 0.05 and *p* < 0.01, respectively.

TGs are hydrolyzed into glycerol and fatty acids during lipolysis, which is associated with cellular energy homeostasis. In the present study, the nano-liposomal BP at 3.125–12.5 μg/mL significantly increased glycerol release at 2.75–50.39% greater than the unloaded nano-liposomal particles (*p* < 0.01) (Figure 2A).

Oil Red O (ORO) reagent was used to stain the lipid droplet after exposure to the nano-liposomal BPs at 3.125 μg/mL. Staining with ORO revealed that the nano-liposomal BPs significantly decreased fat accumulation in the 3T3-L1 cells (Figure 2C), suggesting that the nano-liposomal BPs exhibited lipolysis activity, as indicated by the increase in glycerol release, and inhibited the accumulation of lipid droplets.

These results show that the highest concentration of nano-liposomal BPs moderately stimulated glycerol release (~50%). Since BPs are not therapeutic drugs, it is impossible to make comparisons with the known anti-diabetes drugs, although fewer adverse effects are associated with BPs. Other peptides from protein hydrolysates such as the tripeptide ILL and the quadrapeptide VHVV reportedly promote lipolysis in fat cells [30]. However, the permeability of these peptides could prohibit their use in functional food products. Thus, a liposome carrier was developed to entrap the peptide molecule. The proposed BPs with lipolysis activity were heat-resistant up to 121 °C for >10 min. Moreover, the gastrointestinal proteases pepsin and trypsin had no effect on the lipolysis-stimulating activity of the BPs in vitro [6]. Hence, the nano-liposomal BPs present a potential functional ingredient to prevent obesity.

### 3.5. Proteome Profiling and Differential Proteins Quantification

**Figure A1:**
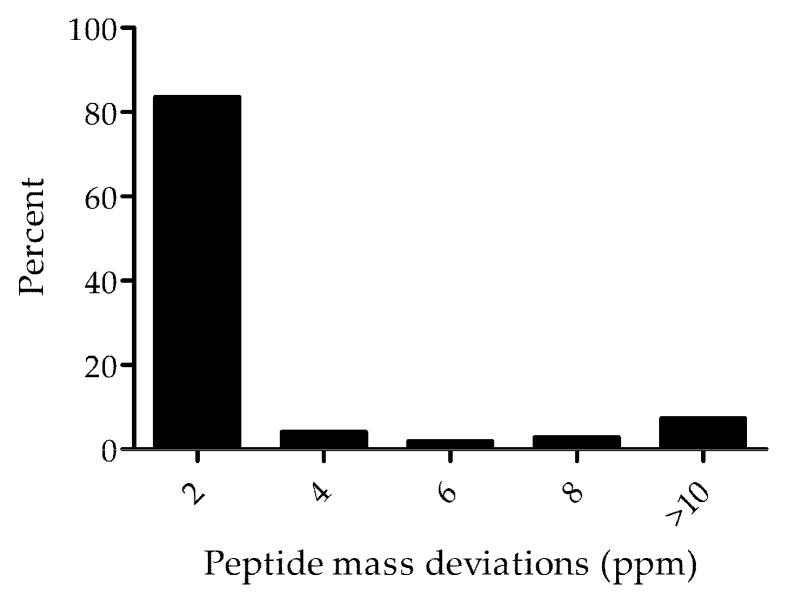
Peptide mass deviation of the measured mass from the theoretical mass of the peptide shown in ppm.

**Figure A2:**
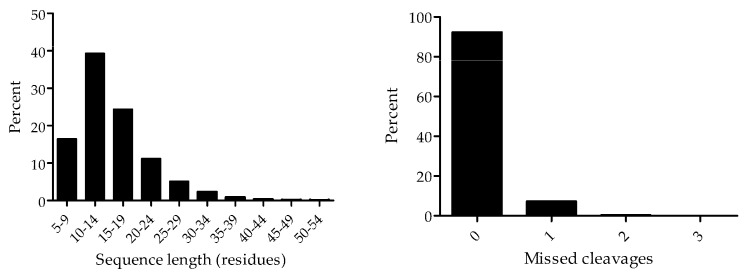
The peptide length (**left**) and missed cleavage site distribution (**right**) of the identified peptides.

**Figure A3:**
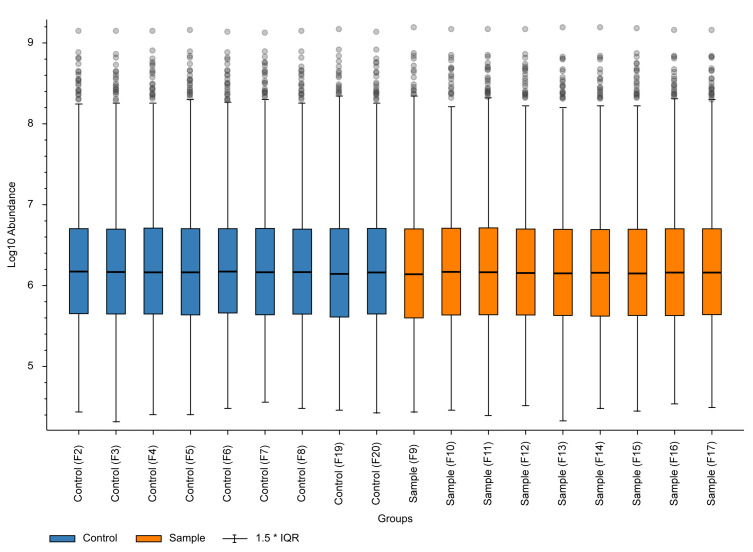
The abundance of protein in the LC-runs (n = 18) of three biological and three instrumental repetitions.

Proteomics analysis was conducted to explore the effect of nano-liposomal BPs on the lipolysis activity of adipocytes. The proteome dataset was used to identify and quantify proteins that were differentially expressed among the sample conditions. QC assessment is highly recommended to avoid any bias in the results of protein expression analysis. The QC results are shown in Appendix A (Figure A1, Figure A2 and Figure A3).

**Figure 3:**
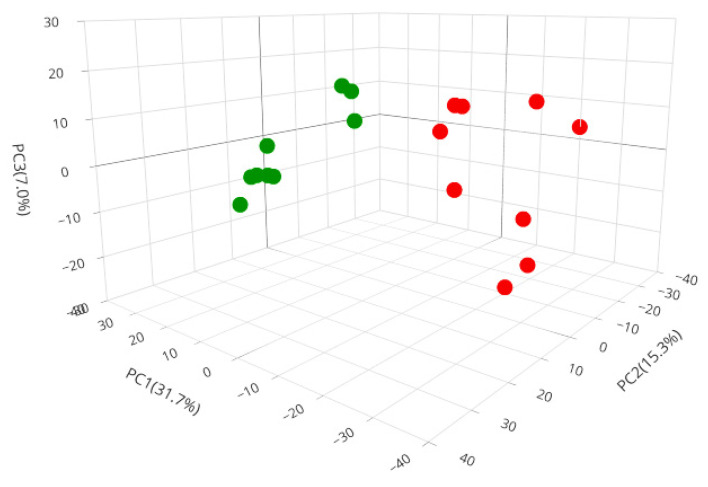
PCA of the proteome data. Nano-liposomal BPs and control conditions are indicated by different colored circles. Nano-liposomal BPs are shown as red circles and the control samples as green circles.

Proteomics is a very sensitive method for protein identification and quantification. Relatively abundant proteins with FDR values less than 0.05 among 18 LC runs (three biological and three instrumental replicates) are considered as significantly differentially expressed. In total, 2418 proteins were successfully identified. To explore the variance in the proteome profiles, PCA analysis was performed by comparing the two sample groups in the 18 LC runs and evaluating the intra- and inter-variations between the treatment and control groups. The PCA of three independent biological replicates and three technical replicates of each group were analyzed. The PCA results demonstrated differences in the proteome profiles (Figure 3).

The nano-liposomal BP groups were clustered in different regions to the control groups. Grouped nearby sample sets had a high correlation in terms of the proteome profile of each condition, but distant sample sets had a lower correlation. The intra-group variations were slightly larger, which may be due to the small sample size or largely uncontrollable factors such as the treatment variation and other factors pre- and post-sample collection. The PCA analysis showed the complete separation of the nano-liposomal BP and control groups, indicating differences in the protein profiles.

**Figure 4:**
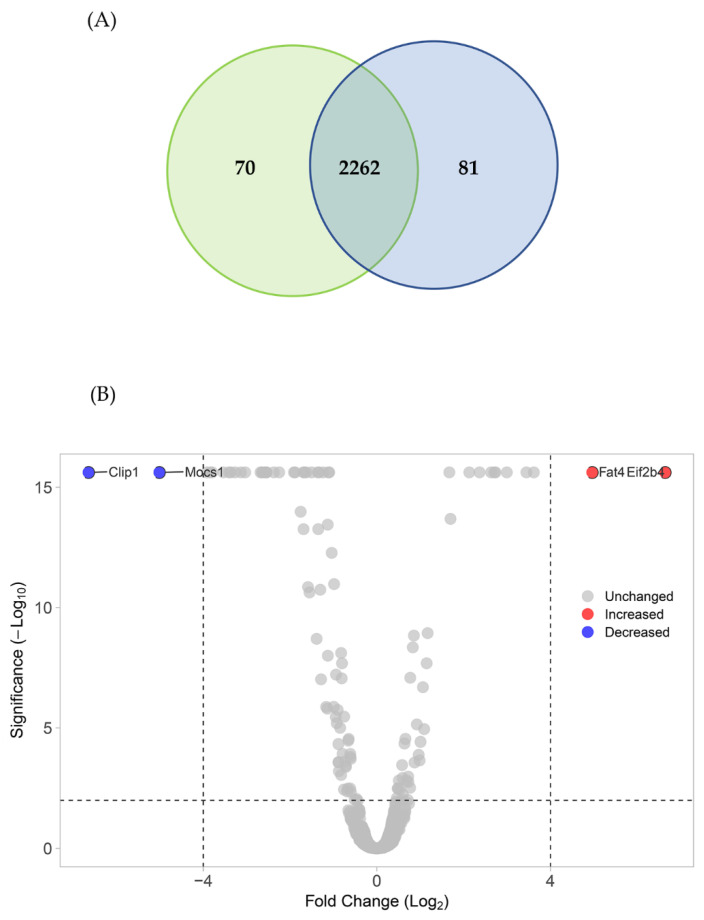
Differences in the proteome profiling visualized by a volcano plot. (**A**) Venn diagram of proteomics analysis. (**B**) The plot shows the negative natural log of the *p*-values and log_2_ fold change of each protein’s change between the nano-liposomal BP and control groups. Statistically significant results (*p* < 0.05) are plotted above the dashed horizontal and vertical line. Proteins significantly up- and downregulated upon treatment with the nano-liposomal BPs are shown as red and blue dots, respectively.

In total, 2418 proteins were successfully identified. According to the Venn diagram (Figure 4A), 70 and 81 proteins were unique to the treatment and control groups, respectively. The details of these proteins are presented in Supplementary Material S2. Interpretation of the proteomics showed that 2262 proteins (five proteins were filtered out due to missing expression values) were affected by the nano-liposomal BPs as shown in the volcano plot in Figure 4B.

As confirmation of the protein quantification results after processing, there should be no difference in the expression levels of the housekeeping proteins. The expression ratio of β-actin (Actbl2; 42 kDa) and histone H2B (H2bc12; 13.9 kDa) between the two experimental groups (n = 9) was 1.043 (*p* = 0.94) and 1.034 (*p* = 0.96), respectively. In addition, there was no significant difference in the total peptide intensity of each injection (LC = 18 runs) (Figure A3). These results suggest that there was no significant difference in the total protein content among the experimental groups. Therefore, the differential expression of the proteins was only due to the nano-liposomal BPs.

The volcano plot revealed four significantly differentially expressed proteins. The global proteins were slightly upregulated (50.18%; for 1135 of 2262 proteins). Specifically, two significantly differentially expressed proteins (*p* < 0.01 and −4 > log2 (fold change) > 4) were downregulated (blue dots, Figure 4) and two were upregulated (red dots, Figure 4).

The downregulated significantly differentially expressed proteins included CAP-Gly domain-containing linker protein 1 (Clip1) and molybdenum cofactor biosynthesis protein 1 (Mocs), whereas the upregulated differentially expressed proteins included protocadherin fat 4 (Fat4) and translation initiation factor eIF-2B subunit delta (Eif2b4). The biological functions of these proteins vary in *Mus musculus*. Clip1 plays an important role in the dynamic regulation of the cytoskeleton and intracellular vesicle trafficking [31]. Mocs is involved in molybdopterin biosynthesis and is required for the activities of molybdenum enzymes [32]. In mouse cells, Fat4 functions in cell–cell adhesion, cell orientation in skeletal morphogenesis, Hippo signaling, and nervous system development [33]. The human ortholog of this protein is FAT atypical cadherin 4, which suppresses tumor growth through the activation of the Hippo signaling pathway [34]. Eif2b4 is a guanine nucleotide exchange factor composed of five different subunits that acts as a major regulator of protein synthesis in living cells [35]. These proteins do not directly, but rather indirectly, interact with proteins in the lipolysis pathway, as determined by proteomics analysis. Notably, the nano-liposomal BPs influenced various biochemical pathways in 3T3-L1 cells other than lipolysis.

### 3.6. Quantification of FAS by ELISA and Western Blotting

**Figure 5:**
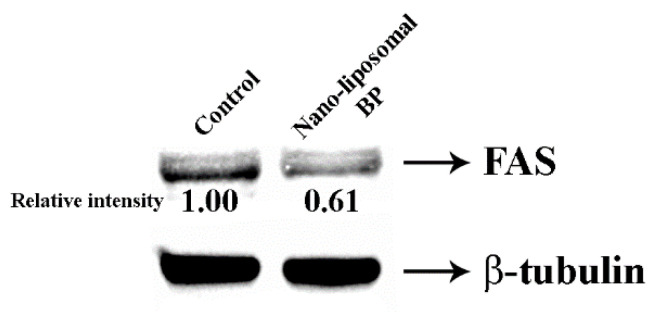
FAS protein expression validation by Western blot analysis with the housekeeping protein, β-tubulin. Proteins from each of the experimental groups were fractionated by SDS-PAGE and transferred to the PVDF membrane. The FAS and β-tubulin were immunodetected using the primary antibody and HRP-secondary antibody.

A colorimetric ELISA was used to assess the influence of BP on FAS expression in 3T3-L1 cells. Cells treated with nanoliposomal BPs had an observable inhibitory effect compared to the control. The abundance of FAS was 17.41 ± 1.17% lower in the experimental group than the control group (*p* ≤ 0.01), indicating that the BPs affected FAS expression. To confirm the reliability of ELISA data, we used Western blot analysis to quantitate FAS protein abundance (Figure 5).

FAS is a crucial enzyme that controls the de novo synthesis of fatty acids, and thus presents a potential anti-obesity target [36]. In addition, FAS has also been associated with homeostatic regulation of feeding behavior and acts directly on appetite control via the nervous system [36]. Therefore, the inhibition of FAS expression with BPs could be used to treat obesity and reduce body weight. The ELISA results and Western blot analysis of FAS expression confirmed the proteomics and lipolysis results. Different biochemical methods were used to assess the expression level of FAS. Although FAS was among the most downregulated proteins, the expression was decreased by 0.019-fold compared to the control group. Hence, molecular docking analysis between FAS and BP was conducted to clarify the possible mechanisms inhibiting the suppression of FAS.

### 3.7. BP-FAS Molecular Docking Analysis

**Figure 6:**
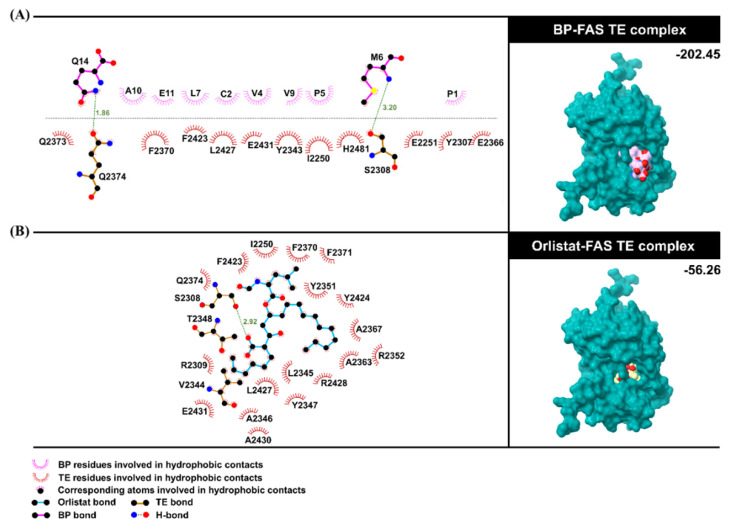
Binding interaction and orientation of (**A**) the focused BP structure with the TE domain of FAS at the active site compared to (**B**) the hydrolyzed orlistat–TE complex. HDOCK scores of both complexes are also shown.

A molecular docking study was used to investigate the BP binding to the FAS TE domain using the HDOCK webserver. The docking result of the BP–FAS TE complex is depicted in Figure 6 and compared to that of orlistat, a well-known anti-obesity drug that inhibits FAS by forming a covalent adduct with the TE domain [37]. Through the N-terminal insertion into the active site of FAS TE, the BP produced an HDOCK score of -202.45 that outperformed the hydrolyzed orlistat (-56.26).

The BP binding was well-stabilized by hydrophobic interactions with the 13 TE residues (i.e., I2250, E2251, Y2307, S2308, Y2343, E2366, F2370, Q2373, Q2374, F2423, L2427, E2431, and H2481). Additionally, the backbone nitrogen of the BP residue M6 formed hydrogen bonding with the hydroxyl group of the catalytic residue S2308, while the amide nitrogen of the C-terminal residue Q14 made a hydrogen bond with the amide oxygen of Q2374. In contrast, only a hydrogen bond with the catalytic residue S2308 was detected in the reference ligand.

## 4. Conclusions

In conclusion, we discovered a novel bioactive peptide (BP) for which the sequence was NH_2_-PCGVPMLTVAEQAQ-CO_2_H. It was chemically synthesized using solid-phase peptide synthesis (SPPS) and successfully encapsulated in a nano-liposomal carrier. The encapsulation efficiency was ~61%, which was lower than expected, presumably due to the disruption of entrapment of the BP via electrostatic interactions between the amino acid side chains and the liposome surface, preventing full loading. Nonetheless, we demonstrated the capacity of the nano-liposomal BP to reduce lipid droplets and increase glycerol release in mouse adipocyte cells without affecting the cell viability. The treatment with the nano-liposomal BP also reduced FAS expression in the cells as shown by Western blotting and ELISA. Proteomics revealed that the nano-liposomal BP influenced various biochemical pathways in the cells in addition to lipolysis. Furthermore, molecular docking analysis also showed the binding interactions between the BP and TE-FAS. Overall, the nano-liposomal BP reported herein could be used as a promising ingredient for functional food products, especially for the prevention of obesity.

## Figures and Tables

**Table 1:** Peptide purity and molecular mass of the peptide NH_2_-PCGVPMLTVAEQAQ-CO_2_H.

Chemical Formula	Theoretical mass ^a^ (Da)	Observed Mass ^b^ (Da)	Mass Deviation ^c^ (%)	Purity (%)
C_61_H_102_N_16_O_20_S_2_	1443.70	1443.60	0.007	89.51%

^a^ The theoretical molecular mass was calculated as the monoisotopic mass of the constituent atoms. ^b^ The observed molecular mass is the monoisotopic mass of the constituent atoms as determined by LC-MS. ^c^ Measurement error of the theorical and observed masses.

## Data Availability

The data presented in this study are available on request from the corresponding author.

## Supplementary Materials

The following supporting information can be downloaded at: https://www.mdpi.com/article/10.3390/foods12040780/s1, Supplementary Material S1: Supplemental method: Peptide synthesis reaction; Figure S1: The 3D structures of (A) FAS TE predicted by AlphaFold2 and (B) BP modeled by PEP-FOLD3; Supplementary Material S2: The details of 2418 proteins.

## Appendix A. QC of Proteomics Data

To qualify the mass accuracy of the peptides, the characteristics of the identified tryptic peptides were evaluated directly. The peptide mass deviation is an important parameter related to mass accuracy in proteomics data. Our results revealed that 83.67% of the identified peptides had a narrow mass deviation (Δm < 2 ppm), as shown in Figure A1.

The peptide mass deviation was also strictly set at 0–20 ppm. The value of a narrow peptide mass deviation acts as a filter that directly reduces the number of false-positive peptides. These results show that the major peptides achieved a good mass accuracy. Sample preparation and tryptic digestion efficiency were investigated. The majority of identified peptides (74.84%) were between 10 and 25 amino acid residues (Figure A2 left). Furthermore, 92.33% of the identified peptides also revealed no missed cleavage peptides (Figure A2, right).

We observed about 8% of the tryptic peptides containing one or more tryptic sites, which corresponded to the properties of the tryptic digestion products. The MS and MS/MS signal from the short peptide length (<6 amino acid residues) and a high number of missed cleavage sites (>3 sites) can influence the protein identification quality.

Normalization of the protein expression values requires datasets to be corrected. To figure out this issue, protein-level normalization was conducted to reduce the non-biological related variations and to ensure reliable results for downstream analysis (Figure A3).

Normalization reduced the variation in the groups and intergroups measured by log protein abundance because the raw data contained missing values, especially for those with relatively low abundance in the cellular system. This is common in most tandem mass spectroscopy techniques due to the randomness in sampling during the MS and MS/MS acquisition. The limitations of these missing expression values can be improved by eliminating or including peptide normalization algorithms and the statistical analysis of all of the quantified proteins.
