# Supplementary material for: Proteomics and Molecular Docking Analyses Reveal the Bio-Chemical and Molecular Mechanism Underlying the Hypolipidemic Activity of Nano-Liposomal Bioactive Peptides in 3T3-L1 Adipocytes
Source: Foods. 2023 Feb 10;12(4):780. doi: 10.3390/foods12040780 (PMC9956075; doi:10.3390/foods12040780)
Supplement: Supplementary file 1 [file foods-12-00780-s001.zip › Supporting material S1.pdf]

Supplementary material S1

Supplemental method: Peptide synthesis reaction

The resin for peptide synthesis (1 g, 1.2 mmol/g) was washed and incubated in roughly 10 mL of N-methylpyrrolidinone (NMP) for 16 hours prior to each reaction. The deprotection solution (50% piperidine in NMP; 4 mL of NMP and 4 mL of piperidine) were added to the resin for 10 minutes with nitrogen gas. The solution was filtered from the resin and the resin was washed using dichloromethane (DCM) and NMP, this process was repeated for a total of four times to prepare the resin for amino acid elongation processes. For elongation processes contain 14 reactions following;

1. To obtain resin linked with L-glutamine, Fmoc-Glutamine-OH (3 eq.) was added diisopropylethylamine (DIPEA) (6 eq.) and NMP (8 mL), and the solution was incubated for 30 seconds. The solution was then added to the resin and the mixture was sparged for 20 minutes with nitrogen gas. The solution was filtered from the resin and washed using NMP and DCM. The deprotecting solution 6 mL of 50% piperidine in NMP and piperidine were added to the resin, and the mixture was mixing for 2 minutes. The solution was filtered from the resin-Q and was washed using NMP and DCM, and the reaction was analyzed for completion reaction using the Kaiser test before Alanine elongation.

2. To obtain resin-QA linked with L-alanine, Fmoc-alanine-OH (3 eq.) was added DIPEA (6 eq.) and NMP (8 mL), and the solution was incubated for 30 seconds. The solution was then added to the resin-Q and the mixture was sparged for 20 minutes with nitrogen gas. The solution was filtered from the resin-Q and washed using NMP and DCM. The deprotecting solution 6 mL of 50% piperidine in NMP and piperidine were added to the resin-Q, and the mixture was mixing for 2 minutes. The solution was filtered from the resin-QA and was washed using NMP and DCM. The reaction was analyzed for completion reaction using the Kaiser test before glutamine elongation.

3. To obtain resin-QAQ linked with L-glutamine, Fmoc-glutamine-OH (3 eq.) was added DIPEA (6 eq.) and NMP (8 mL), and the solution was incubated for 30 seconds. The solution was then added to the resin-QA and the mixture was sparged for 20 minutes with nitrogen gas. The solution was filtered from the resin-QAQ and washed using NMP and DCM. The deprotecting solution, 6 mL of 50% piperidine in NMP and piperidine were added to the resin-QA, and the mixture was mixing for 2 minutes. The solution was filtered from the resin-QAQ and was washed using NMP and DCM. The reaction was analyzed for completion reaction using the Kaiser test before glutamic acid elongation.

4. To obtain resin-QAQE linked with L-glutamic acid, Fmoc-glutamic acid-OH (3 eq.) was added DIPEA (6 eq.) and NMP (8 mL), and the solution was incubated for 30 seconds. The solution was then added to the resin-QAQ and the mixture was sparged for 20 minutes with nitrogen gas. The solution was filtered from the resin-QAQE and washed using NMP and DCM. The deprotecting solution, 6 mL of 50% piperidine in NMP and piperidine were added to the resin-QAQ, and the mixture was mixing for 2 minutes. The solution was filtered from the resin-QAQE and was washed using NMP and DCM. The reaction was analyzed for completion reaction using the Kaiser test before alanine elongation.

5. To obtain resin-QAQEA linked with L-alanine, Fmoc-alanine-OH (3 eq.) was added DIPEA (6 eq.) and NMP (8 mL), and the solution was incubated for 30 seconds. The solution was then added to the resin-QAQE and the mixture was sparged for 30 minutes with nitrogen gas. The solution was filtered from the resin-QAQE and washed using NMP and DCM. The deprotecting solution, 6 mL of 50% piperidine in NMP and piperidine were added to the resin-QAQE, and the mixture was mixing

for 2 minutes. The solution was filtered from the resin-QAQEA and was washed using NMP and DCM. The reaction was analyzed for completion reaction using the Kaiser test before valine elongation.

6. To obtain resin-QAQEAV linked with L-valine, Fmoc-alanine-OH (3 eq.) was added DIPEA (6 eq.) and NMP (8 mL), and the solution was incubated for 30 seconds. The solution was then added to the resin-QAQEA and the mixture was sparged for 30 minutes with nitrogen gas. The solution was filtered from the resin-QAQEA and washed using NMP and DCM. The deprotecting solution, 6 mL of 50% piperidine in NMP and piperidine were added to the resin-QAQEA, and the mixture was mixing for 2 minutes. The solution was filtered from the resin-QAQEAV and was washed using NMP and DCM. The reaction was analyzed for completion reaction using the Kaiser test before threonine elongation.

7. To obtain resin-QAQEAVT linked with L-threonine, Fmoc-threonine-OH (3 eq.) was added DIPEA (6 eq.) and NMP (8 mL), and the solution was incubated for 30 seconds. The solution was then added to the resin-QAQEAV and the mixture was sparged for 30 minutes with nitrogen gas. The solution was filtered from the resin-QAQEAV and washed using NMP and DCM. The deprotecting solution, 6 mL of 50% piperidine in NMP and piperidine were added to the resin-QAQEAV, and the mixture was mixing for 2 minutes. The solution was filtered from the resin-QAQEAVT and was washed using NMP and DCM. The reaction was analyzed for completion reaction using the Kaiser test before leucine elongation.

8. To obtain resin-QAQEAVTL linked with L-leucine, Fmoc-threonine-OH (3 eq.) was added DIPEA (6 eq.) and NMP (8 mL), and the solution was incubated for 30 seconds. The solution was then added to the resin-QAQEAVT and the mixture was sparged for 50 minutes with nitrogen gas. The solution was filtered from the resin-QAQEAVT and washed using NMP and DCM. The deprotecting solution, 6 mL of 50% piperidine in NMP and piperidine were added to the resin-QAQEAVT, and the mixture was mixing for 2 minutes. The solution was filtered from the resin-QAQEAVTL and was washed using NMP and DCM. The reaction was analyzed for completion reaction using the Kaiser test before methionine elongation.

9. To obtain resin-QAQEAVTLM linked with L-methionine, Fmoc-methionine-OH (3 eq.) was added DIPEA (6 eq.) and NMP (8 mL), and the solution was incubated for 30 seconds. The solution was then added to the resin-QAQEAVTL and the mixture was sparged for 50 minutes with nitrogen gas. The solution was filtered from the resin-QAQEAVTL and washed using NMP and DCM. The deprotecting solution, 8 mL of 50% piperidine in NMP and piperidine were added to the resin-QAQEAVTL (2 times), and the mixture was mixing for 2 minutes. The solution was filtered from the resin-QAQEAVTLM and was washed using NMP and DCM. The reaction was analyzed for completion reaction using the Kaiser test before proline elongation.

10. To obtain resin-QAQEAVTLMP linked with L-proline, Fmoc-proline-OH (3 eq.) was added DIPEA (6 eq.) and NMP (8 mL), and the solution was incubated for 30 seconds. The solution was then added to the resin-QAQEAVTLM and the mixture was sparged for 60 minutes with nitrogen gas. The solution was filtered from the resin-QAQEAVTLM and washed using NMP and DCM. The deprotecting solution, 8 mL of 50% piperidine in NMP and piperidine were added to the resin-QAQEAVTLM (2 times), and the mixture was mixing for 2 minutes. The solution was filtered from the resin-QAQEAVTLMP and was washed using NMP and DCM. The reaction was analyzed for completion reaction using the Kaiser test before valine elongation.

11. To obtain resin-QAQEAVTLMPV linked with L-valine, Fmoc-proline-OH (3 eq.) was added DIPEA (6 eq.) and NMP (8 mL), and the solution was incubated for 30 seconds. The solution

was then added to the resin-QAQEAVTLMP and the mixture was sparged for 40 minutes with nitrogen gas (2 times). The solution was filtered from the resin-QAQEAVTLMP and washed using NMP and DCM. The deprotecting solution, 8 mL of 50% piperidine in NMP and piperidine were added to the resin-QAQEAVTLMP (2 times), and the mixture was mixing for 5 minutes. The solution was filtered from the resin-QAQEAVTLMPV and was washed using NMP and DCM. The reaction was analyzed for completion reaction using the Kaiser test before glycine elongation.

12. To obtain resin-QAQEAVTLMPVG linked with L-cysteine, Fmoc-proline-OH (3 eq.) was added DIPEA (6 eq.) and NMP (8 mL), and the solution was incubated for 30 seconds. The solution was then added to the resin-QAQEAVTLMPV and the mixture was sparged for 40 minutes with nitrogen gas (2 times). The solution was filtered from the resin-QAQEAVTLMPV and washed using NMP and DCM. The deprotecting solution, 8 mL of 50% piperidine in NMP and piperidine were added to the resin-QAQEAVTLMPV (2 times), and the mixture was mixing for 5 minutes. The solution was filtered from the resin-QAQEAVTLMPVG and was washed using NMP and DCM. The reaction was analyzed for completion reaction using the Kaiser test before cysteine elongation.

13. To obtain resin-QAQEAVTLMPVGC linked with L-cysteine, Fmoc-cysteine-OH (3 eq.) was added DIPEA (6 eq.) and NMP (8 mL), and the solution was incubated for 30 seconds. The solution was then added to the resin-QAQEAVTLMPVG and the mixture was sparged for 40 minutes with nitrogen gas (2 times). The solution was filtered from the resin-QAQEAVTLMPVG and washed using NMP and DCM. The deprotecting solution, 8 mL of 50% piperidine in NMP and piperidine were added to the resin-QAQEAVTLMPVG (2 times), and the mixture was mixing for 5 minutes. The solution was filtered from the resin-QAQEAVTLMPVGC and was washed using NMP and DCM. The reaction was analyzed for completion reaction using the Kaiser test before proline elongation.

14. To obtain resin-QAQEAVTLMPVGCP linked with L-cysteine, Fmoc-cysteine-OH (3 eq.) was added DIPEA (6 eq.) and NMP (8 mL), and the solution was incubated for 30 seconds. The solution was then added to the resin-QAQEAVTLMPVGC and the mixture was sparged for 40 minutes with nitrogen gas (2 times). The solution was filtered from the resin-QAQEAVTLMPVGC and washed using NMP and DCM. The deprotecting solution, 8 mL of 50% piperidine in NMP and piperidine were added to the resin-QAQEAVTLMPVGC (2 times), and the mixture was mixing for 5 minutes. The solution was filtered from the resin-QAQEAVTLMPVGCP and was washed using NMP and DCM. The reaction was analyzed for completion reaction using the Kaiser test before cleavage synthesized peptides from the resin.

Final synthetic peptides were deprotected and cleaved from the resin by cleavage cocktail containing trifluoroacetic acid (TFA)/ethanedithiol (EDT)/ triisopropylsilane (TIPS)/ water = 93:2.5:2.5:2 (v/v) at room temperature. The synthetic peptide was precipitated with cold diethyl ether by 1:10 (v/v) and collected the crude synthetic peptide pelleted by centrifugation at 10000g, 4°C for 30 minutes. The synthesized peptide product was cleaved from resin as above in order to verify identity using LC-MS.

Supplemental figure: Computational studies of BP binding to FAS thioesterase

(A)

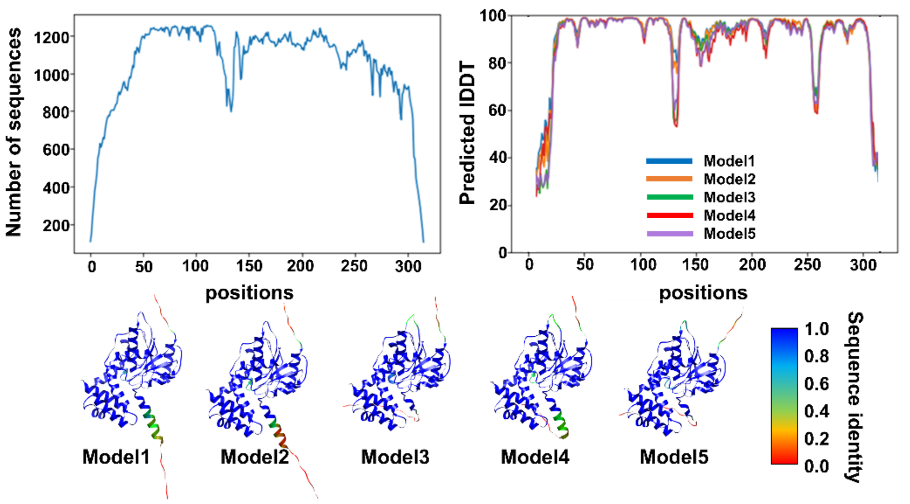

(B)

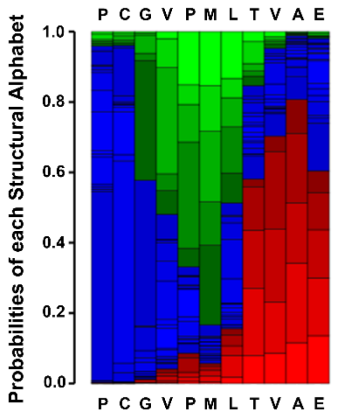

Model	sOPEP	gdt	q	tm
Model1	-15.24	0.72	0.53	0.50
Model2	-14.40	0.73	0.54	0.52
Model3	-14.24	0.74	0.58	0.51
Model4	-14.19	0.74	0.55	0.50
Model5	-14.08	0.72	0.52	0.49

sOPEP : The coarse grained energy of PEP-FOLD3
gdt : The predicted Global Distance Test Total Score
q : The predicted Qmean score
tm : The predicted TMscore

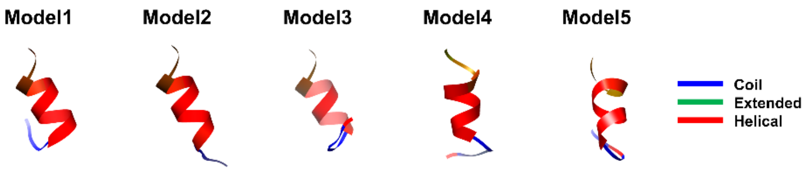

Figure S1. The 3D structures of (A) FAS TE predicted by AlphaFold2 and (B) BP modeled by PEP-FOLD3.
